# TMS‐Derived Neurophysiological Measures of Corticospinal Excitability, Intracortical Excitability, and Interhemispheric Interactions Associated With Post‐Stroke Motor Recovery: A Systematic Review

**DOI:** 10.1111/ejn.70698

**Published:** 2026-10-04

**Authors:** Gabrielly Santos Pereira, Marcelo Lourenço da Silva, Abrahão Fontes Baptista, João Eduardo de Araujo

**Affiliations:** ^1^ Neuropsychobiology and Motor Control Laboratory, Department of Health Sciences, Ribeirão Preto Medical School University of São Paulo Ribeirão Preto São Paulo Brazil; ^2^ Laboratory of Neuroscience, Neuromodulation and Study of Pain (LANNED) Federal University of Alfenas (UNIFAL‐MG) Alfenas Minas Gerais Brazil; ^3^ School of Physiotherapy Federal University of Rio de Janeiro Rio de Janeiro Rio de Janeiro Brazil

**Keywords:** corticospinal excitability, motor evoked potential, motor recovery, neurorehabilitation, rTMS, stroke, transcranial magnetic stimulation

## Abstract

Motor recovery after stroke is associated with changes in corticospinal integrity, intracortical excitability, and interhemispheric interactions. Transcranial magnetic stimulation (TMS) provides neurophysiological measures of these processes, but their relationship with motor recovery across randomized controlled trials remains uncertain. This systematic review synthesized evidence from randomized controlled trials investigating TMS‐derived neurophysiological measures associated with motor recovery following TMS‐based neuromodulation after stroke. PubMed/MEDLINE, Scopus, and Embase were searched for studies published between January 1, 2016, and July 10, 2026. Eligible trials enrolled adults with ischemic or hemorrhagic stroke, assessed at least one TMS‐derived neurophysiological measure, and reported a motor or functional outcome. Study selection followed PRISMA 2020, risk of bias was assessed using Cochrane RoB 2, and certainty of evidence was evaluated using GRADE. Owing to methodological heterogeneity, findings were synthesized narratively. The search identified 1441 records; after removal of 519 duplicates, 922 records were screened, 55 full‐text reports were assessed, and 16 randomized controlled trials were included. Motor evoked potential (MEP) amplitude, MEP status, and resting motor threshold were the most frequently reported measures. Several trials found that changes in corticospinal excitability accompanied improvements in upper‐limb function, activities of daily living, gait, or balance. One trial found greater motor gains among participants with preserved baseline MEPs; however, the contemporary randomized‐trial evidence is insufficient to establish MEP status as a predictor of differential response to neuromodulation. In contrast, short‐interval intracortical inhibition, cortical silent period, and interhemispheric inhibition were assessed in very few trials, whereas intracortical facilitation was not directly evaluated in any eligible RCT. Most studies raised some risk‐of‐bias concerns, and one study had high overall risk. Certainty was moderate for clinical motor recovery, low for MEP‐ and motor‐threshold‐based measures, and very low for intracortical and interhemispheric measures. Overall, MEP‐derived measures and resting motor threshold were the most frequently reported neurophysiological domains, whereas baseline MEP status was evaluated in only one stratified trial. Standardized TMS protocols are needed to clarify their monitoring, prognostic, and treatment‐response value.

## Graphical abstract

Our systematic review identified 16 randomized controlled trials investigating TMS‐derived neurophysiological measures associated with post‐stroke motor recovery. We summarize the evidence for corticospinal, intracortical, and interhemispheric biomarkers, evaluate their association with clinical recovery, and discuss their potential prognostic and treatment‐monitoring value.

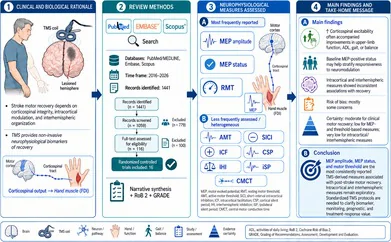

**Abbreviations**
ADL: activities of daily living
AMT: active motor threshold
ARAT: Action Research Arm Test
BBS: Berg Balance Scale
BBT: Box and Block Test
BI: Barthel Index
cAMT: contralesional active motor threshold
CEAI: corticomotor excitability asymmetry index
CMCT: central motor conduction time
CSP: cortical silent period
FIM: Functional Independence Measure
FMA: Fugl‐Meyer Assessment
FMA‐LE: Fugl‐Meyer Assessment–Lower Extremity
FMA‐MI: Fugl‐Meyer Assessment motor impairment domain
FMA‐UE: Fugl‐Meyer Assessment–Upper Extremity
GRADE: Grading of Recommendations Assessment, Development and Evaluation
iAMT: ipsilesional active motor threshold
ICF: intracortical facilitation
IHA: interhemispheric asymmetry
IHI: interhemispheric inhibition
iSP: ipsilateral silent period
iTBS: intermittent theta‐burst stimulation
LF‐rTMS: low‐frequency repetitive transcranial magnetic stimulation
LICI: long‐interval intracortical inhibition
M1: primary motor cortex
MEP: motor evoked potential
MRC: Medical Research Council
mRS: modified Rankin Scale
NHPT: Nine‐Hole Peg Test
NIHSS: National Institutes of Health Stroke Scale
PASS: Postural Assessment Scale for Stroke Patients
PMd: dorsal premotor cortex
PRISMA: Preferred Reporting Items for Systematic Reviews and Meta‐Analyses
PROSPERO: International Prospective Register of Systematic Reviews
RCT: randomized controlled trial
RMT: resting motor threshold
rTMS: repetitive transcranial magnetic stimulation
SICI: short‐interval intracortical inhibition
SMA: supplementary motor area
TBS: theta‐burst stimulation
TIS: Trunk Impairment Scale
TMS: transcranial magnetic stimulation
TMS–EEG: transcranial magnetic stimulation combined with electroencephalography
TUG: Timed Up and Go Test
WMFT: Wolf Motor Function Test

## 1 Introduction

Stroke remains one of the leading causes of death and long‐term disability worldwide and continues to impose a substantial burden on individuals, families, and healthcare systems. In 2021, stroke accounted for approximately 7.3 million deaths and 160.5 million disability‐adjusted life‐years globally, underscoring its persistent public health impact. Although advances in acute stroke management have improved survival, motor impairment remains highly prevalent and is a major determinant of long‐term dependence and reduced quality of life. Restoration of motor function therefore remains one of the primary goals of post‐stroke neurorehabilitation (Winstein et al. 2016; Feigin et al. 2024).

Motor recovery after stroke depends largely on neuroplasticity and the capacity of the central nervous system to reorganize surviving neural networks. Following cerebral injury, disruption of corticospinal pathways, altered intracortical inhibition, and changes in interhemispheric interactions contribute to persistent motor deficits, although the functional role of contralesional activity may vary according to residual corticospinal integrity and impairment severity (Ward 2017; Di Pino et al. 2014).

Among noninvasive neuromodulation techniques, repetitive transcranial magnetic stimulation (rTMS) has emerged as one of the most extensively investigated therapeutic approaches for promoting post‐stroke motor recovery. Depending on stimulation frequency and target, rTMS can increase excitability within the ipsilesional motor network or reduce contralesional cortical activity, with the aim of modulating maladaptive interhemispheric interactions and facilitating corticospinal output. Multiple randomized controlled trials have demonstrated improvements in upper‐limb function, gait performance, and activities of daily living following rTMS, although substantial interindividual variability remains (Winstein et al. 2016; Lefaucheur et al. 2020).

Beyond its therapeutic application, transcranial magnetic stimulation (TMS) provides a unique opportunity to quantify neurophysiological changes associated with recovery. Single‐ and paired‐pulse TMS protocols provide complementary information about different levels of motor‐system physiology. Resting and active motor thresholds (RMT and AMT) reflect the excitability of cortical and corticospinal elements required to generate a motor response, whereas motor evoked potential (MEP) amplitude reflects the net output of the corticospinal system and is influenced by cortical recruitment, corticospinal conduction, spinal excitability, and peripheral factors. MEP latency and central motor conduction time (CMCT) provide information about corticospinal conduction. Paired‐pulse measures probe intracortical circuitry: Short‐interval intracortical inhibition (SICI) is primarily associated with GABA_A_‐mediated inhibitory mechanisms, intracortical facilitation (ICF) reflects facilitatory intracortical interactions with an important glutamatergic contribution, and long‐interval intracortical inhibition (LICI) is more closely associated with GABA_B_‐mediated inhibition. The cortical silent period (CSP) also reflects inhibitory mechanisms, particularly GABA_B_‐related processes during voluntary contraction, whereas interhemispheric inhibition (IHI) and the ipsilateral silent period (iSP) provide information about transcallosal inhibitory interactions between motor cortices. Together, these measures characterize complementary aspects of corticospinal integrity, intracortical excitability, and interhemispheric network function that may change during post‐stroke recovery (Rossini et al. 2015; Groppa et al. 2012).

Among these neurophysiological measures, MEP status has particular prognostic relevance because the presence or absence of an evoked response provides information about functional corticospinal tract integrity. This principle underlies biomarker‐based prediction algorithms such as PREP and PREP2, in which MEP status contributes to early stratification of upper‐limb recovery potential, particularly in patients with more severe initial motor impairment. MEP status has been associated with upper‐limb motor recovery, particularly during the acute and early subacute stages after stroke (Stinear 2017; Stinear et al. 2012; Byblow et al. 2015; Yen et al. 2023; Jordan et al. 2026). Accordingly, consensus recommendations from the Stroke Recovery and Rehabilitation Roundtable support the inclusion of baseline MEP status as a biomarker for prognostic assessment and participant stratification in stroke recovery research (Boyd et al. 2017). However, the prognostic value of MEP status is context and stage dependent and should not be equated with prediction of differential treatment response; recent evidence indicates that baseline MEP status does not necessarily predict responsiveness to upper‐limb training in individuals with chronic moderate‐to‐severe post‐stroke hemiparesis (King et al. 2025). MEP amplitude, latency, and recruitment characteristics provide additional information on corticospinal excitability and conduction and have also been associated with motor impairment and recovery after stroke (Rossini et al. 2015; Groppa et al. 2012; McDonnell and Stinear 2017). Similarly, motor thresholds reflect the excitability of neural elements contributing to corticospinal output, whereas paired‐pulse paradigms such as SICI and ICF assess inhibitory and facilitatory intracortical circuits involved in post‐stroke cortical physiology (Rossini et al. 2015; Groppa et al. 2012). These measures have therefore been investigated as mechanistic and prognostic markers of recovery, although their ability to predict individual responsiveness to rehabilitation or neuromodulation remains insufficiently established (Ward 2017; Stinear 2017; Boyd et al. 2017). Importantly, prognostic value should be distinguished from treatment‐response prediction: A prognostic measure is associated with outcome irrespective of treatment, whereas a treatment‐response modifier identifies differential benefit from an intervention according to baseline physiological status.

Despite the growing body of evidence, the neurophysiological changes accompanying motor improvement during rTMS‐based post‐stroke rehabilitation remain incompletely understood. Previous systematic reviews have primarily focused on the clinical efficacy of rTMS or compared stimulation protocols, whereas relatively little attention has been given to the neurophysiological measures that accompany functional improvement. Furthermore, the consistency of TMS‐derived measures across randomized controlled trials has not been comprehensively synthesized, limiting the interpretation of their prognostic, mechanistic, and treatment‐monitoring value in clinical neurorehabilitation.

Therefore, the present systematic review aimed to synthesize the contemporary randomized‐trial evidence regarding TMS‐derived neurophysiological measures associated with motor recovery following TMS‐based neuromodulation interventions in adults after stroke. Specifically, this review evaluated changes in measures including RMT, AMT, MEP, SICI, ICF, LICI, CSP, and IHI and examined whether these changes accompanied, correlated with, or predicted motor and functional outcomes reported in randomized controlled trials.

Unlike previous reviews that synthesized TMS measures across observational and interventional designs, the present review focused exclusively on randomized controlled trials and examined whether treatment‐related or baseline TMS‐derived measures accompanied, correlated with, or stratified motor recovery following TMS‐based neuromodulation.

## 2 Methods

This systematic review was conducted in accordance with the Preferred Reporting Items for Systematic Reviews and Meta‐Analyses (PRISMA 2020) statement and aimed to synthesize the contemporary randomized‐trial evidence regarding TMS‐derived neurophysiological measures associated with motor recovery following stroke. The review protocol was prospectively registered in the International Prospective Register of Systematic Reviews (PROSPERO; Registration Number CRD420261439852).

### 2.1 Eligibility Criteria

Studies were selected according to predefined eligibility criteria. Randomized controlled trials involving adults (≥ 18 years) diagnosed with ischemic or hemorrhagic stroke at any stage of recovery (acute, subacute, or chronic) were considered eligible.

Studies were required to investigate rTMS, theta‐burst stimulation (TBS), or combined neuromodulation protocols in which rTMS or TBS constituted the central transcranial stimulation component and to report at least one TMS‐derived neurophysiological measure together with a validated clinical motor or functional outcome. Paired associative stimulation paradigms were not excluded solely on the basis of associative timing. Combined protocols were eligible when they incorporated a train‐based rTMS or TBS component together with an eligible TMS‐derived neurophysiological outcome. In contrast, cortico‐cortical or cerebello‐motor paired associative stimulation paradigms without an eligible train‐based rTMS or TBS component were outside the intervention scope of this review. Peripheral magnetic stimulation alone was not considered an eligible intervention because the review focused on neurophysiological effects associated with transcranial stimulation. However, combined protocols incorporating both transcranial rTMS or TBS and repetitive peripheral magnetic stimulation were eligible when the central stimulation component and at least one eligible TMS‐derived neurophysiological measure were reported. Eligible neurophysiological measures included RMT, AMT, MEP amplitude, latency, or status, CMCT, SICI, ICF, LICI, CSP, iSP, and IHI.

For the purposes of this review, MEP status was defined as MEP positive or MEP negative according to whether a reproducible MEP could be elicited by TMS over the relevant primary motor cortex and recorded from the target muscle using surface electromyography, based on the study‐specific criteria used in each trial.

Clinical outcomes included validated measures of motor impairment and functional recovery, such as the Fugl‐Meyer Assessment, Action Research Arm Test, Wolf Motor Function Test, Box and Block Test, Nine‐Hole Peg Test, Barthel Index, Modified Barthel Index, gait and balance assessments, grip strength, and other standardized measures of motor performance.

Observational studies, case reports, case series, reviews, editorials, conference abstracts, animal studies, pediatric studies, and studies that did not evaluate TMS‐derived neurophysiological measures together with motor outcomes were excluded. Studies were excluded when TMS was used solely to determine motor threshold for stimulation dosing, localize the stimulation target, or guide intervention delivery, without a TMS‐derived neurophysiological measure being analyzed either as a baseline prognostic or treatment‐response variable or as a longitudinal outcome.

### 2.2 Information Sources and Search Strategy

A comprehensive literature search was conducted in PubMed/MEDLINE, Embase, and Scopus. Studies published from January 1, 2016, to July 10, 2026, were eligible. The final search was performed on July 10, 2026. Search strategies combined controlled vocabulary (MeSH terms where applicable) and free‐text terms related to stroke, TMS, rTMS, corticospinal excitability, MEPs, RMT and AMT, intracortical inhibition, ICF, IHI, CSP, and motor recovery. Boolean operators (“AND” and “OR”) were applied according to the syntax of each database to optimize sensitivity and specificity. Only studies published in English or Portuguese were considered. All retrieved references were exported to EndNote (Clarivate Analytics), where duplicate records were removed using both automated and manual procedures. The search was designed primarily to identify conventional rTMS and TBS interventions and did not specifically target associative stimulation paradigms lacking an eligible train‐based rTMS or TBS component. The complete electronic search strategies for all databases are provided in the Supporting Information.

The 2016 lower date limit was selected to provide a focused synthesis of contemporary randomized evidence using current rTMS/TBS intervention and neurophysiological assessment approaches. Earlier literature has been synthesized in previous reviews; however, because methodological standards, stimulation protocols, and TMS acquisition procedures have evolved substantially, the present review was designed to evaluate the most recent decade of randomized evidence.

### 2.3 Study Selection

Following duplicate removal, two reviewers (G.S.P. and M.L.d.S.) independently screened the titles and abstracts of all retrieved records according to the predefined eligibility criteria. The full texts of potentially eligible reports were subsequently assessed by the same reviewers. Disagreements at either stage were resolved through discussion and consensus, with consultation of a third reviewer when necessary (J.E.d.A.). The entire selection process was documented using a PRISMA 2020 flow diagram.

### 2.4 Data Extraction

Data were independently extracted by two reviewers (G.S.P. and M.L.d.S.). Disagreements were resolved by consensus or consultation with a third reviewer (J.E.d.A.). Information extracted from each study included the first author, year of publication, study design, sample characteristics, stroke type and recovery stage, stimulation protocol, cortical target, stimulation frequency and intensity, number of stimulation sessions, comparator group, neurophysiological measures assessed, clinical motor outcomes, follow‐up period, and principal findings. For TMS‐derived neurophysiological assessments, additional methodological information was extracted whenever reported, including the target muscle, hemisphere assessed, stimulation intensity used for MEP acquisition, number of trials, motor state during recording (rest or active contraction), EMG recording procedures, threshold definition, and timing of post‐intervention assessment. The extracted information was cross‐checked before inclusion in the narrative synthesis.

### 2.5 Risk of Bias Assessment

Risk of bias in the included randomized controlled trials was assessed using the Cochrane RoB 2 tool. Risk of bias was evaluated across the domains of bias arising from the randomization process, deviations from intended interventions, missing outcome data, measurement of outcomes, and selection of reported results.

Each study was classified as presenting low risk of bias, some concerns, or high risk of bias according to the recommendations of the RoB 2 framework. Two reviewers performed the assessments independently (G.S.P. and M.L.d.S.), and disagreements were resolved by consensus or consultation with a third reviewer (J.E.d.A.).

### 2.6 Certainty of Evidence

The certainty of the evidence was assessed using the Grading of Recommendations Assessment, Development and Evaluation (GRADE) approach. The certainty of evidence for each outcome domain was evaluated considering risk of bias, inconsistency, indirectness, imprecision, and publication bias and was classified as high, moderate, low, or very low certainty.

The outcome domains comprised clinical motor recovery, MEP‐derived measures, motor thresholds, intracortical inhibition and facilitation, and interhemispheric measures.

### 2.7 Data Synthesis

Because substantial methodological heterogeneity was identified across the included studies with respect to stroke stage, stimulation protocols, cortical targets, neurophysiological assessment methods, intervention characteristics, and clinical outcome measures, a quantitative meta‐analysis was not considered appropriate. Therefore, the findings were synthesized narratively.

The results were organized according to the principal neurophysiological domains, including corticospinal excitability (RMT, AMT, MEP, and CMCT), intracortical inhibition and facilitation (SICI, ICF, LICI, and CSP), and interhemispheric inhibition (IHI and iSP). Whenever possible, neurophysiological findings were interpreted alongside clinical motor outcomes to determine whether changes in TMS‐derived measures were reported to accompany, correlate with, or predict functional improvements following neuromodulation interventions.

## 3 Results

### 3.1 Study Selection

The database search identified 1441 records from PubMed/MEDLINE, Scopus, and Embase. After removal of 519 duplicate records, 922 records remained for title and abstract screening. During this stage, 867 records were excluded because they did not meet the predefined eligibility criteria. The main reasons for exclusion were the absence of a randomized controlled design, lack of an eligible TMS‐derived neurophysiological measure analyzed as a baseline prognostic or stratification variable or as a longitudinal outcome, absence of an eligible motor outcome, use of TMS solely for dosing or target localization, and failure to report separate data for adult stroke participants.

After title and abstract screening, 55 full‐text reports were assessed for eligibility. Of these, 39 reports were excluded. The main reasons for exclusion were absence of a randomized controlled design, lack of eligible longitudinal TMS‐derived neurophysiological measures, absence of motor outcomes, and use of TMS only as an intervention parameter or targeting method without an eligible baseline or longitudinal neurophysiological analysis.

Finally, 16 randomized controlled trials met all eligibility criteria and were included in the systematic review. The study selection process is summarized in the PRISMA 2020 flow diagram shown in Figure 1.

**FIGURE 1:**
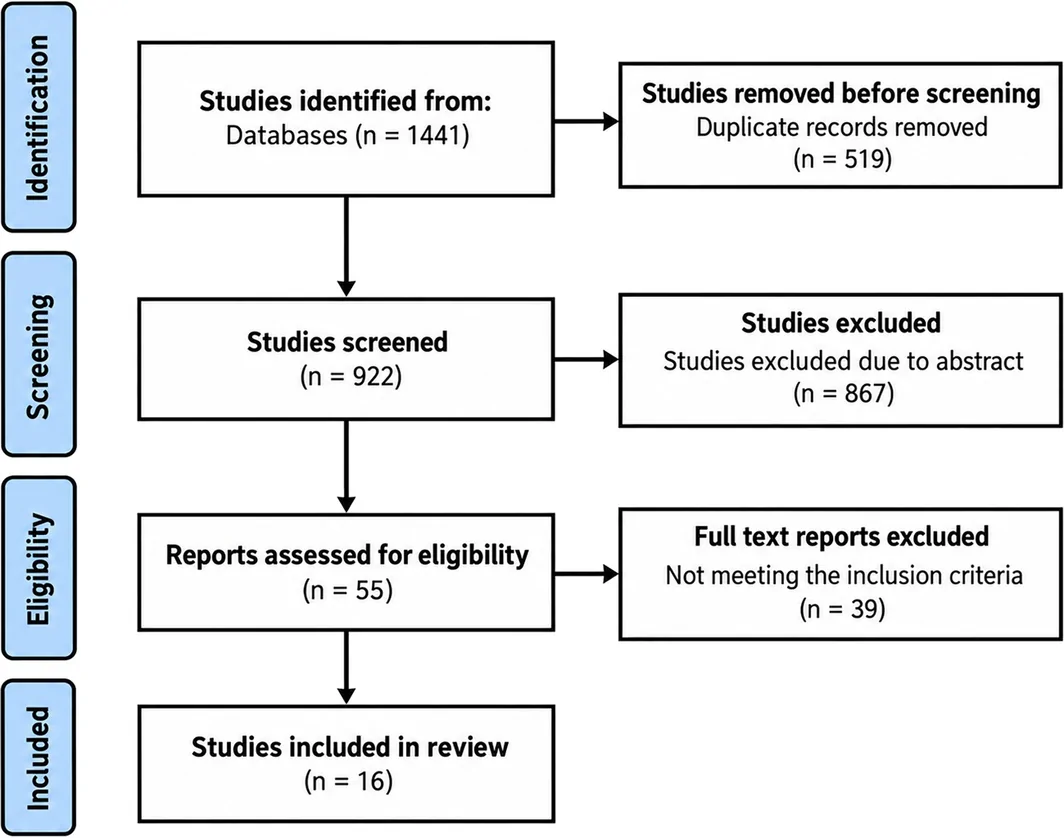
PRISMA 2020 flow diagram of the study selection process.

### 3.2 Characteristics of Included Studies

The 16 included randomized controlled trials were published between 2016 and 2026 and enrolled adult participants with ischemic or hemorrhagic stroke. The studies included participants across different stages of recovery, including acute, subacute, and chronic stroke. Five trials included participants in the acute or early subacute stage, several focused on subacute stroke, and two investigated chronic stroke populations. The definitions and time windows used to classify stroke stage varied across studies.

Most studies focused on upper‐limb motor recovery, including assessments of hand function, dexterity, grip strength, and upper‐extremity impairment (Ackerley et al. 2016; Du et al. 2016; Lüdemann‐Podubecká et al. 2016; El‐Tamawy et al. 2019; Meng et al. 2020; Chen et al. 2021; Luk et al. 2022; Lv et al. 2023; Liang et al. 2024; Qian et al. 2024; Valovičová et al. 2024; Li et al. 2026; Guan et al. 2017). Lower‐limb function, gait, or balance outcomes were assessed in fewer studies, particularly those by Huang et al. (2018), Liao et al. (2024), and Zhao et al. (2025). One included trial combined central rTMS with repetitive peripheral magnetic stimulation, illustrating that combined central–peripheral protocols were eligible when a transcranial stimulation component and TMS‐derived neurophysiological outcomes were reported (Liang et al. 2024).

Sample sizes ranged from 10 to 100 participants, and the number of treatment sessions, stimulation intensities, comparator conditions, rehabilitation programs, and follow‐up periods differed substantially across trials. Intervention dose also varied considerably across studies, including differences in stimulation frequency, intensity, number of sessions, and duration of concomitant rehabilitation. These characteristics are summarized in Table 1.

**TABLE 1:** Characteristics and principal findings of the included randomized controlled trials.

Study	Participants, *n*/stroke stage	Stimulation target	Intervention and dose	TMS‐derived measures	Neurophysiological assessment summary	Clinical outcomes	Principal neurophysiological findings
Ackerley et al. (2016)	18/chronic	Ipsilesional M1	iTBS, 600 pulses at 90% AMT, followed by 45‐min individualized upper‐limb PT; 10 consecutive weekdays	MEP, RMT, CEAI	Bilateral FDI; RMT and MEP stimulus–response curves obtained with surface EMG	ARAT, FMA‐UE	A shift toward more balanced corticomotor excitability asymmetry was associated with sustained upper‐limb functional improvement.
Du et al. (2016)	69/acute–subacute	Ipsilesional or contralesional M1	Ipsilesional 3 Hz, 80%–90% RMT, or contralesional 1 Hz, 110%–120% RMT; 1200 pulses/session; 5 daily sessions + standard PT	RMT, MEP amplitude, MEP latency	APB; bilateral cortical excitability assessment	FMA, MRC, NIHSS, BI, mRS	Reduced RMT, increased MEP amplitude, and shorter MEP latency accompanied motor improvement after active rTMS.
Lüdemann‐Podubecká et al. (2016)	10/subacute	Contralesional dorsal PMd	1‐Hz rTMS, 900 pulses at 110% RMT; one real and one sham session in randomized crossover design	MEP, RMT, CSP, iSP	Bilateral FDI; 20 MEPs at 110% RMT obtained at rest	JTT, BBT	Hand function improved after real rTMS despite no significant changes in MEP, RMT, CSP, or iSP.
Guan et al. (2017)	42 randomized; 27 completed follow‐up/acute	Ipsilesional M1	5‐Hz rTMS, 120% RMT, 1000 pulses/session; 10 daily sessions + rehabilitation	RMT	Bilateral ADM; RMT measured from both hemispheres	NIHSS, BI, FMA‐UE, FMA‐LE, mRS	No significant between‐group RMT difference despite greater early clinical motor improvement after active rTMS.
Huang et al. (2018)	38 randomized; 34 completed/subacute (10–90 days)	Contralesional leg motor cortex	1 Hz, 120% AMT, 900 pulses/session; 15 sessions over 3 weeks, followed by 45‐min PT	AMT, MEP amplitude, MEP latency	Quadriceps/rectus femoris representation; double‐cone coil	TUG, FMA‐LE, PASS, BI	Neurophysiological measures did not differ significantly between groups and did not parallel recovery of ambulation.
El‐Tamawy et al. (2019)	40/subacute	Contralesional M1	1‐Hz rTMS at 90% AMT, 20 min/session; 10 sessions over 2 weeks + conventional rehabilitation	cAMT, iAMT, interhemispheric AMT difference	FDI; excitability assessed from both hemispheres	FMA‐UE, grip strength	Increased contralesional AMT, reduced ipsilesional AMT, and reduced interhemispheric AMT asymmetry accompanied upper‐limb improvement.
Meng et al. (2020)	28/subacute	Bilateral M1	Contralesional 1‐Hz rTMS, 1200 pulses at 100% RMT, followed by ipsilesional iTBS, 1200 pulses at 80% RMT; 10 sessions over 2 weeks	MEP amplitude, MEP latency	MEPs assessed before and after treatment; detailed acquisition parameters incompletely reported	FMA‐UE, BI	Increased ipsilesional MEP amplitude and shorter latency were associated with greater upper‐limb motor improvement.
Chen et al. (2021)	100/acute–subacute	Bilateral M1	Coupled ipsilesional 10‐Hz and contralesional 1‐Hz rTMS; 4‐week intervention	RMT, MEP amplitude, CMCT	Cortical excitability assessed 24 h before and after intervention; detailed MEP acquisition parameters NR	FMA, FMA‐UE, NIHSS, ADL, mRS	Greater increase in MEP amplitude and reduction in RMT accompanied motor recovery; CMCT showed no consistent change.
Luk et al. (2022)	24 randomized; 22 completed/subacute	Contralesional M1	1‐Hz rTMS, 90% RMT, 1200 pulses/session; 10 weekday sessions + motor‐task practice	MEP amplitude, IHA	Bilateral FDI; 10 MEPs at 120% baseline RMT; neuronavigated assessment	FMA‐UE, ARAT, BBT	Increased ipsilesional MEP amplitude and reduced interhemispheric asymmetry correlated with upper‐limb recovery.
Lv et al. (2023)	72/subacute	Contralesional M1	1‐Hz rTMS, 80% RMT, 1200 pulses/session; groups received 10, 20, or 30 sessions + conventional rehabilitation	MEP amplitude	APB; MEP amplitude reassessed after successive treatment blocks	FMA‐UE, WMFT	Reduced contralesional MEP amplitude accompanied improvement; the most consistent clinical and physiological effects occurred after 20 sessions.
Liang et al. (2024)	45/subacute (< 3 months)	Ipsilesional M1 + paretic C7 nerve root	Central rTMS: 5 Hz, 80% RMT, 1200 pulses; paired rPMS delivered 20 ms later; 20 daily sessions over 4 weeks + PT	SICI, RMT, MEP amplitude, MEP latency	APB; MEP at 120% RMT; SICI at 2‐ms ISI; recordings at rest	FMA‐UE, BI, FCA	SICI decreased after combined stimulation, but its change was not significantly associated with functional gains; RMT and MEP measures were less responsive.
Liao et al. (2024)	36/subacute	Ipsilesional M1 or contralesional cerebellum	M1‐iTBS, cerebellar‐iTBS, or sham; 1200 pulses/day at 80% RMT; 15 sessions over 3 weeks + PT	RMT, MEP amplitude, CSP	FDI; corticospinal assessment from M1; RMT determined in relaxed muscle	FMA‐MI, BBS, TIS	MEP amplitude increased after both active interventions, whereas RMT and CSP remained unchanged; clinical recovery was greater after cerebellar iTBS.
Qian et al. (2024)	88/subacute	Contralesional M1	1‐Hz rTMS, 100% contralesional RMT, 1200 pulses/session, 20 min; 20 sessions over 4 weeks + rehabilitation	MEP status, RMT, MEP amplitude, CMCT, IHA	Bilateral APB; 10 MEPs at 120% baseline RMT; participants stratified by baseline MEP status	FMA, NIHSS, BI	MEP‐positive participants showed greater motor gains together with more favorable changes in RMT, MEP amplitude, CMCT, and interhemispheric asymmetry.
Valovičová et al. (2024)	26/acute ischemic stroke	Ipsilesional M1	10‐Hz rTMS, 100% RMT, 1500 pulses/session; 5 sessions	MEP amplitude, MEP latency, CMCT	FDI hotspot; figure‐of‐eight coil; pre/post electrophysiological assessment	NIHSS, FMA‐UE, FMA‐LE, BI, FIM	Clinical improvement occurred without significant changes in MEP amplitude, latency, or CMCT.
Zhao et al. (2025)	69/approximately 1 month post‐stroke; stage not explicitly classified	Ipsilesional SMA or M1	10‐Hz rTMS, 100% RMT, 2400 pulses/session, 20 min; 20 sessions over 4 weeks + routine rehabilitation	MEP amplitude, MEP latency	Figure‐of‐eight coil; TMS‐MEP assessed before and after treatment; target muscle/details NR	FMA‐UE, FMA‐LE, BBS	Both targets increased MEP amplitude and shortened latency; neurophysiological changes did not differ significantly between targets despite greater clinical gains in the SMA group.
Li et al. (2026)	16/chronic severe stroke	Contralesional dorsal PMd or ipsilesional M1	5‐Hz rTMS, 2100 pulses/session, generally at 90% AMT; 2 sessions/week for 6 weeks (12 sessions) + 1‐h upper‐limb training	MEP, IHI	MRI‐guided neuronavigation; bilateral EDC for cortical/IHI measures; detailed EMG acquisition	FMA‐UE, WMFT	MEP responses increased after treatment, whereas IHI responses were heterogeneous and showed limited association with motor recovery; no clear target superiority.

Abbreviations: ADL, activities of daily living; AMT, active motor threshold; ARAT, Action Research Arm Test; BBS, Berg Balance Scale; BBT, Box and Block Test; BI, Barthel Index; cAMT, contralesional active motor threshold; CEAI, corticomotor excitability asymmetry index; CMCT, central motor conduction time; CSP, cortical silent period; FIM, Functional Independence Measure; FMA, Fugl‐Meyer Assessment; FMA‐LE, Fugl‐Meyer Assessment–Lower Extremity; FMA‐MI, Fugl‐Meyer Assessment motor impairment domain; FMA‐UE, Fugl‐Meyer Assessment–Upper Extremity; iAMT, ipsilesional active motor threshold; IHA, interhemispheric asymmetry; IHI, interhemispheric inhibition; iSP, ipsilateral silent period; iTBS, intermittent theta‐burst stimulation; JTT, Jebsen–Taylor Hand Function Test; LF‐rTMS, low‐frequency repetitive transcranial magnetic stimulation; M1, primary motor cortex; MEP, motor evoked potential; MRC, Medical Research Council muscle strength scale; mRS, modified Rankin Scale; NIHSS, National Institutes of Health Stroke Scale; PASS, Postural Assessment Scale for Stroke Patients; PMd, dorsal premotor cortex; RMT, resting motor threshold; SICI, short‐interval intracortical inhibition; SMA, supplementary motor area; TIS, Trunk Impairment Scale; TUG, Timed Up and Go Test; WMFT, Wolf Motor Function Test.

### 3.3 TMS‐Derived Neurophysiological Measures

The most frequently assessed TMS‐derived neurophysiological measures were MEPs and RMT. MEP‐based measures were reported in several studies as amplitude, latency, presence or absence, or a general index of corticospinal excitability (Ackerley et al. 2016; Du et al. 2016; Lüdemann‐Podubecká et al. 2016; Meng et al. 2020; Chen et al. 2021; Luk et al. 2022; Lv et al. 2023; Liang et al. 2024; Qian et al. 2024; Valovičová et al. 2024; Li et al. 2026; Huang et al. 2018; Liao et al. 2024; Zhao et al. 2025). RMT was also commonly used as a marker of corticospinal excitability, particularly in trials investigating rTMS‐induced modulation of ipsilesional or contralesional motor cortex excitability (Ackerley et al. 2016; Du et al. 2016; Lüdemann‐Podubecká et al. 2016; Chen et al. 2021; Liang et al. 2024; Qian et al. 2024; Guan et al. 2017; Liao et al. 2024).

Less frequently reported measures included AMT, CSP, SICI, and IHI; neither ICF nor LICI was directly assessed as an eligible neurophysiological outcome in any included trial. El‐Tamawy et al. (2019) assessed AMT measures in both hemispheres and explored their relationship with upper‐limb motor recovery. Lüdemann‐Podubecká et al. (2016) assessed MEP, RMT, CSP, and iSP as markers of corticospinal excitability, long‐lasting intracortical inhibition, and IHI. Liang et al. (2024) specifically included SICI as a neurophysiological outcome, whereas Li et al. (2026) incorporated MEP and IHI measures in participants with chronic severe stroke.

Overall, MEP amplitude, MEP status, and RMT were the most consistently used neurophysiological measures across the included studies. Measures of intracortical inhibition and IHI were assessed in only a small subset of studies and were obtained using heterogeneous protocols.

The methodological procedures used to obtain TMS‐derived measures also varied substantially across trials. Studies differed in the target muscle and hemisphere assessed, motor‐threshold definition, stimulation intensity used for neurophysiological testing, number of accepted responses, resting versus active recording conditions, EMG processing procedures, and timing of post‐intervention assessment. Several reports did not provide complete information for one or more of these parameters. A detailed cross‐study comparison is provided in Table S1. No single acquisition characteristic consistently distinguished studies reporting significant neurophysiological–behavioral associations from those reporting null or dissociated findings. Positive associations were most frequently observed for longitudinal MEP‐derived measures in studies of upper‐limb recovery, but similar MEP‐based assessments also yielded absent or discordant neurophysiological changes in other trials.

### 3.4 Clinical and Motor Outcomes

Clinical outcomes included the Fugl‐Meyer Assessment, Action Research Arm Test, Box and Block Test, Nine‐Hole Peg Test, Wolf Motor Function Test, grip strength, gait and balance measures, Barthel Index, Modified Barthel Index, modified Rankin Scale, and National Institutes of Health Stroke Scale (NIHSS). Upper‐limb motor outcomes were especially common, with Fugl‐Meyer upper‐extremity scores and task‐based hand function tests reported in several trials (Ackerley et al. 2016; Lüdemann‐Podubecká et al. 2016; El‐Tamawy et al. 2019; Meng et al. 2020; Luk et al. 2022; Lv et al. 2023; Liang et al. 2024; Qian et al. 2024; Li et al. 2026). Lower‐limb and balance outcomes were assessed using measures such as the Berg Balance Scale, Fugl‐Meyer lower‐extremity score, gait assessments, the Trunk Impairment Scale, and the Functional Ambulation Categories (Huang et al. 2018; Liao et al. 2024; Zhao et al. 2025).

Across the included trials, active neuromodulation was generally associated with improvement in at least one motor or functional outcome, although the magnitude and specificity of these effects varied according to stroke stage, stimulation protocol, cortical target, comparator condition, and concomitant rehabilitation. Evidence was predominantly derived from upper‐limb outcomes, whereas lower‐limb, gait, and balance recovery were investigated in substantially fewer trials.

The relationship between clinical improvement and TMS‐derived neurophysiological change was not uniform. A subset of trials reported parallel improvements in motor performance and corticospinal measures, particularly changes in MEP amplitude, motor threshold, or interhemispheric asymmetry (Du et al. 2016; Meng et al. 2020; Chen et al. 2021; Luk et al. 2022; Qian et al. 2024). In contrast, other trials demonstrated clinical improvement without corresponding changes in MEP amplitude, latency, motor threshold, CSP, iSP, or CMCT (Lüdemann‐Podubecká et al. 2016; Valovičová et al. 2024; Guan et al. 2017; Huang et al. 2018). Thus, the contemporary randomized‐trial evidence supports a partial rather than consistent correspondence between behavioral recovery and individual TMS‐derived measures.

Interpretation of these clinical improvements also requires consideration of the comparator condition. In most trials, rTMS or TBS was delivered in addition to conventional rehabilitation, physical therapy, or motor‐task practice, and comparator groups generally received sham stimulation, rehabilitation alone, or an alternative stimulation protocol. Accordingly, improvement within an active‐treatment group cannot be attributed exclusively to neuromodulation. The clearest evidence for an additional treatment effect was provided by trials demonstrating greater motor improvement after active stimulation than after sham or rehabilitation‐matched control conditions (Du et al. 2016; Meng et al. 2020; Chen et al. 2021). In contrast, other trials reported clinical improvement without a clear between‐group neurophysiological or functional advantage (Lüdemann‐Podubecká et al. 2016; Valovičová et al. 2024; Guan et al. 2017; Huang et al. 2018). These findings indicate that the contribution of neuromodulation should be interpreted as an adjunctive effect superimposed on spontaneous recovery and motor rehabilitation rather than as an isolated driver of recovery.

### 3.5 Synthesis by Neurophysiological Domain

Across the 16 included randomized controlled trials, TMS‐derived neurophysiological measures were useful for characterizing changes accompanying post‐stroke motor rehabilitation. The strongest and most frequently reported evidence involved MEP amplitude, MEP status, and RMT, which were used as markers of corticospinal excitability in trials of rTMS, iTBS, combined stimulation protocols, and rehabilitation‐based interventions (Ackerley et al. 2016; Du et al. 2016; Meng et al. 2020; Chen et al. 2021; Luk et al. 2022; Lv et al. 2023; Qian et al. 2024; Guan et al. 2017; Liao et al. 2024; Zhao et al. 2025).

Evidence for measures of intracortical inhibition and IHI was more limited. SICI was directly assessed by Liang et al. (2024), CSP and iSP were reported by Lüdemann‐Podubecká et al. (2016), and IHI‐related measures were included in Li et al. (2026). These measures may provide potentially important mechanistic information about inhibitory control, cortical reorganization, and interhemispheric balance, but their use was inconsistent across studies and the assessment protocols were heterogeneous.

Taken together, the findings suggest that TMS‐derived neurophysiological measures may be valuable tools for monitoring neuroplasticity and motor recovery after stroke. However, the evidence does not support interpreting all TMS‐derived measures as interchangeable biomarkers. MEP measures and RMT primarily reflect corticospinal excitability, whereas SICI, CSP, ICF, and IHI provide complementary information about intracortical and interhemispheric mechanisms. Therefore, a multimodal TMS assessment approach may provide a more comprehensive characterization of post‐stroke motor recovery than any single neurophysiological measure alone.

The distribution of neurophysiological measures across the 16 included trials was markedly uneven (Table 2). At least one MEP‐derived measure was assessed in 14 trials (87.5%), whereas RMT was evaluated as a neurophysiological outcome in eight (50.0%), MEP latency in six (37.5%), and interhemispheric asymmetry indices in four (25.0%). CMCT was assessed in three trials (18.8%), AMT and CSP in two trials each (12.5%), and MEP status, SICI, iSP, and IHI in only one trial each (6.3%). No eligible randomized trial directly assessed neither ICF nor LICI as a longitudinal or stratification outcome. This distribution highlights the concentration of the randomized‐trial evidence around corticospinal output measures and the scarcity of evidence for intracortical and interhemispheric measures.

**TABLE 2:** Quantitative evidence map of TMS‐derived neurophysiological measures across the included randomized controlled trials.

TMS‐derived measure	Trials, *n* (%)	Overall relationship with motor recovery	Certainty/interpretation
Any MEP‐derived measure	14/16 (87.5%)	Most frequently investigated domain. Several trials reported longitudinal changes accompanying motor improvement, but clinically meaningful recovery also occurred without corresponding MEP changes.	Low certainty. Most developed measure for monitoring corticospinal change, but not a validated surrogate of recovery.
MEP latency	6/16 (37.5%)	Shorter latency accompanied improvement in some trials, whereas others found no significant change despite clinical recovery.	Low certainty. Complementary measure of corticospinal conduction.
MEP status (present/absent)	1/16 (6.3%)	MEP‐positive participants showed greater motor gains in one stratified RCT.	Low certainty. Promising for baseline stratification, but insufficient evidence for prediction of differential treatment response.
RMT	8/16 (50.0%)	Reductions accompanied improvement in some studies, but several trials showed clinical gains without significant RMT changes.	Low certainty. Useful index of corticospinal excitability; limited as an isolated recovery marker.
AMT/hemispheric AMT measures	2/16 (12.5%)	Changes in hemispheric excitability accompanied improvement in some participants, but evidence was sparse.	Low certainty/sparse evidence.
CMCT	3/16 (18.8%)	Findings were inconsistent; favorable changes were reported in one stratified trial, whereas other studies showed little or no change.	Low to very low certainty.
SICI	1/16 (6.3%)	SICI decreased after combined central–peripheral stimulation, but the change was not significantly associated with functional gain.	Very low certainty. Exploratory mechanistic measure.
CSP	2/16 (12.5%)	Clinical improvement was not consistently accompanied by CSP changes.	Very low certainty.
iSP	1/16 (6.3%)	Clinical improvement occurred without a significant iSP change.	Very low certainty.
IHI	1/16 (6.3%)	IHI responses were heterogeneous and showed limited association with recovery.	Very low certainty. Mechanistically relevant but insufficient for clinical interpretation.
Interhemispheric asymmetry indices	4/16 (25.0%)	More balanced hemispheric excitability accompanied improvement in several studies, although the indices and acquisition procedures differed substantially.	Low to very low certainty. Promising mechanistic domain requiring standardization.
ICF	0/16 (0%)	No eligible RCT directly assessed ICF as a longitudinal or stratification outcome.	No direct randomized‐trial evidence in the present review.
LICI	0/16 (0%)	No eligible RCT directly assessed LICI as a longitudinal or stratification outcome.	No direct randomized‐trial evidence in the present review.

*Note:* Percentages use the 16 included randomized controlled trials as the denominator. Categories overlap because individual trials assessed more than one TMS‐derived measure.

Thus, the direction of neurophysiological findings could not be explained by a single stimulation or acquisition characteristic. Studies reporting significant associations and those reporting clinical–neurophysiological dissociation overlapped considerably with respect to stroke stage, cortical target, stimulation frequency, treatment duration, and the type of rehabilitation provided. Given this clinical and methodological heterogeneity, together with the low or very low certainty of evidence for most neurophysiological domains, the available randomized‐trial evidence does not currently support the use of any single TMS‐derived measure for individual‐level prediction of motor outcome or differential treatment response.

### 3.6 Risk of Bias

The risk of bias assessment is summarized in Figure 2. Overall, the included randomized controlled trials were predominantly judged as presenting some concerns regarding overall risk of bias, whereas one study was classified as having high overall risk of bias. No study fulfilled all criteria to be classified as having a low overall risk of bias.

**FIGURE 2:**
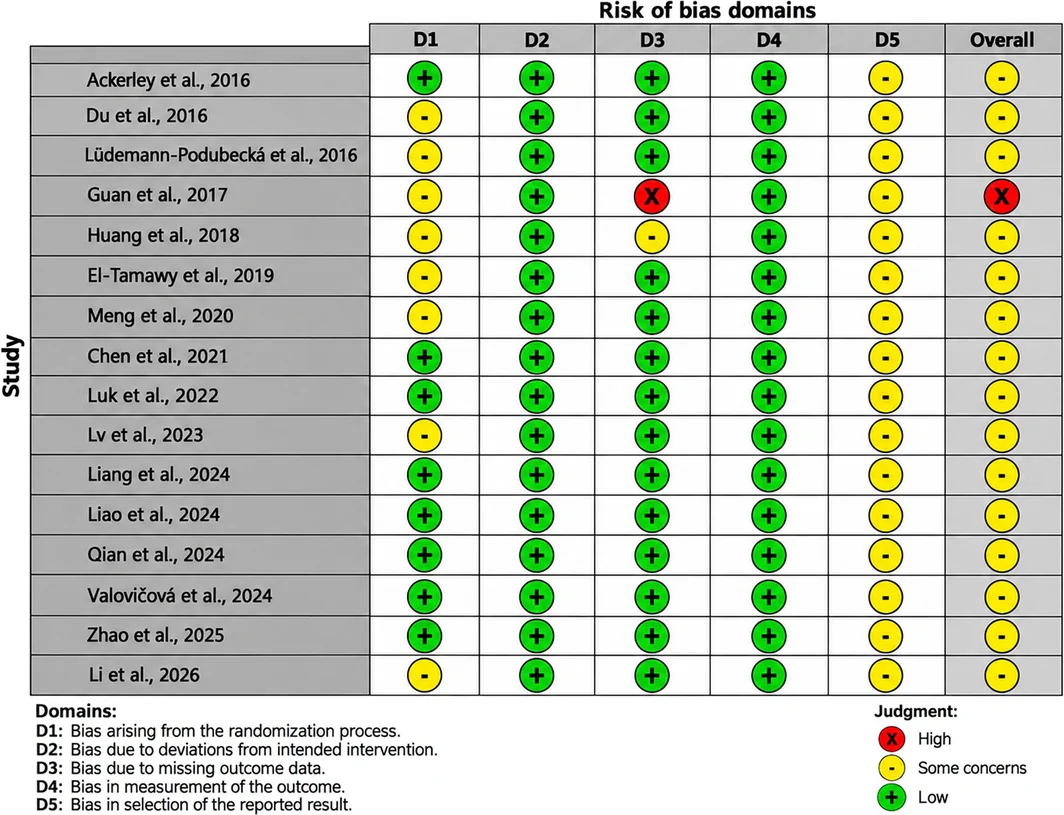
Risk of bias assessment of the included randomized controlled trials using the Cochrane RoB 2 tool.

Risk of bias was assessed for the principal TMS‐derived neurophysiological outcome at the first post‐intervention assessment, considering the effect of assignment to the intervention. The main methodological limitation identified across the included studies was bias in the selection of the reported result (Domain 5). Most trials were judged as having some concerns in this domain because no prospectively registered protocol or prespecified statistical analysis plan was available, making it difficult to exclude selective outcome reporting.

Regarding the randomization process (Domain 1), most studies were judged as having either low risk of bias or some concerns. In several trials, although randomization was reported, insufficient information regarding sequence generation or allocation concealment prevented a judgment of low risk.

The remaining domains generally demonstrated a low risk of bias. Most studies adequately adhered to the intended interventions (Domain 2), had limited missing outcome data (Domain 3), and used validated clinical scales together with objective TMS‐derived neurophysiological measures, resulting in a low risk of bias for outcome measurement (Domain 4). The only study classified as having high overall risk of bias had substantial loss to follow‐up, increasing the risk of bias due to missing outcome data.

The evidence was predominantly affected by some concerns, particularly regarding prespecification and selective reporting, with one trial judged at high overall risk of bias.

### 3.7 Certainty of Evidence

The certainty of evidence for clinical motor recovery was rated as moderate. Most randomized trials reported improvement in at least one motor or functional outcome after active neuromodulation or neuromodulation combined with rehabilitation. Certainty was downgraded primarily because of inconsistency arising from substantial heterogeneity in stroke stage, baseline impairment, stimulation target, treatment dose, comparator conditions, concurrent rehabilitation, and clinical outcome measures.

The certainty of evidence for MEP‐derived measures and motor thresholds was rated as low. MEP amplitude was the most frequently investigated longitudinal neurophysiological measure and accompanied clinical improvement in several trials; however, other studies reported clinical recovery without corresponding MEP changes. Acquisition procedures also varied considerably with respect to target muscle, hemisphere assessed, stimulation intensity, recording state, number of trials, and assessment timing. Motor‐threshold findings were similarly inconsistent, with treatment‐related changes observed in some trials but absent in others.

Evidence for baseline MEP status was also rated as low. Only one randomized trial directly stratified treatment effects according to MEP status, limiting confidence in its value as a treatment‐response modifier. Although preserved MEPs are biologically and prognostically relevant as indicators of residual corticospinal integrity, the current randomized‐trial evidence is insufficient to establish MEP status as a predictor of differential response to neuromodulation.

The certainty of evidence for intracortical inhibitory and facilitatory measures was rated as very low. SICI, CSP, and related measures were investigated in very few trials, acquisition protocols were heterogeneous, and neurophysiological changes did not consistently correspond to functional improvement. No eligible randomized trial directly assessed neither ICF nor LICI as a longitudinal or stratification outcome.

Evidence for interhemispheric measures was rated as low to very low. Interhemispheric asymmetry, iSP, and IHI were assessed infrequently and with heterogeneous methods, and their relationships with motor recovery were inconsistent. Overall, these measures remain mechanistically informative but insufficiently supported for individual‐level clinical prediction or treatment selection.

## 4 Discussion

This systematic review synthesized randomized controlled evidence on TMS‐derived neurophysiological measures associated with motor recovery following TMS‐based neuromodulation after stroke. Three main findings emerged. First, MEP‐derived measures, particularly MEP amplitude, were the most frequently investigated, whereas baseline MEP status was examined in only one stratified randomized trial but had specific prognostic relevance. Second, changes in clinical performance did not consistently parallel changes in corticospinal excitability, indicating that conventional TMS measures capture only part of the neurophysiological processes underlying recovery. Third, evidence for motor thresholds, intracortical inhibition, and interhemispheric measures was sparse, methodologically heterogeneous, and insufficient to support their use as established prognostic or treatment‐response biomarkers. These findings indicate that TMS‐derived measures should be interpreted according to their specific physiological and clinical role rather than as interchangeable indicators of recovery. Consensus recommendations similarly emphasize that candidate biomarkers should be defined according to whether they are intended to characterize biological state, estimate prognosis, monitor recovery, demonstrate target engagement, or identify differential treatment response (Boyd et al. 2017).

Importantly, throughout this review, “motor recovery” refers to observed improvement in motor or functional outcomes over time and should not be interpreted as recovery attributable specifically to rTMS or TBS. Treatment‐related effects can be inferred more directly only from between‐group comparisons against sham or rehabilitation‐matched control conditions, whereas within‐group improvement may also reflect spontaneous biological recovery and concurrent rehabilitation.

Among the available measures, MEP‐derived variables appear most useful for characterizing corticospinal integrity and longitudinal changes in corticospinal output. However, their interpretation depends on the purpose for which they are used. Baseline MEP status primarily provides information on residual corticospinal pathway integrity and recovery potential, whereas longitudinal changes in MEP amplitude may serve as physiological monitoring measures. In contrast, neither measure should automatically be interpreted as a mechanistic mediator of behavioral recovery or as a predictor of differential response to neuromodulation. This distinction is particularly important because several included trials showed clinical improvement without corresponding changes in MEP amplitude, latency, threshold, or inhibitory measures.

Nevertheless, MEP amplitude should not be interpreted as a direct or isolated measure of cortical plasticity. The MEP reflects the combined influence of cortical neuronal recruitment, corticospinal tract conduction, spinal motor‐neuron excitability, peripheral conduction, background muscle activation, coil positioning, stimulation intensity, and moment‐to‐moment variability. Its amplitude therefore represents the output of a distributed corticospinal system rather than the activity of a single cortical circuit. Bestmann and Krakauer (2015) emphasized that an increase or decrease in MEP amplitude cannot, by itself, establish the mechanism responsible for a behavioral change. This distinction is particularly relevant after stroke, when lesions may affect cortical, subcortical, and descending pathways differently (Bestmann and Krakauer 2015).

The imperfect correspondence between neurophysiological and behavioral changes across the included trials reinforces this interpretation. Several studies reported clinically meaningful motor improvement without parallel changes in MEP amplitude, latency, motor threshold, CSP, or iSP, whereas others observed neurophysiological changes that were not accompanied by clearly superior clinical effects. This dissociation suggests that behavioral recovery may depend on mechanisms extending beyond the corticospinal pathways directly sampled by conventional TMS, including premotor, supplementary motor, parietal, cerebellar, reticulospinal, and broader sensorimotor networks, as well as compensatory motor strategies.

Complementary evidence from associative stimulation paradigms also suggests that post‐stroke motor networks can be modulated through temporally coupled interactions between distributed motor regions. For example, cerebello‐motor paired associative stimulation has been investigated in a randomized sham‐controlled trial as a means of modifying motor‐network plasticity after stroke, although such paradigms were outside the intervention scope of the present review (Rosso et al. 2022).

The broader TMS literature supports this heterogeneity. A meta‐analysis of post‐stroke TMS studies found reduced excitability of the affected M1 relative to the unaffected hemisphere and healthy controls but also considerable variability related to stroke stage, impairment severity, muscle examined, and neurophysiological protocol (McDonnell and Stinear 2017). Therefore, an intervention‐related increase in MEP amplitude may indicate greater recruitment of preserved corticospinal pathways in some patients, whereas the absence of such an increase does not exclude clinically meaningful recovery mediated by alternative networks.

A particularly relevant finding concerns baseline MEP status. Unlike MEP amplitude, which quantifies the size of an evoked response, MEP status refers to the presence or absence of a reproducible motor response elicited by stimulation of the relevant M1 representation and recorded from the target muscle. MEP‐positive status therefore provides physiological evidence that a functional corticospinal connection between the stimulated motor cortex and the recorded muscle can still be demonstrated. Qian et al. (2024) reported that MEP‐positive participants experienced greater motor gains and more favorable changes in RMT, MEP amplitude, CMCT, and interhemispheric asymmetry after low‐frequency rTMS than MEP‐negative participants. This result is consistent with prospective studies showing that corticospinal tract integrity contributes substantially to the prediction of upper‐limb recovery. The prognostic relevance of MEP status is also reflected in the PREP and PREP2 algorithms. In the original PREP approach, clinical examination was combined with TMS‐derived MEP status and diffusion‐weighted imaging to estimate upper‐limb recovery potential (Stinear et al. 2012). PREP2 subsequently simplified this framework by sequentially integrating the SAFE score, age, MEP presence or absence, and stroke severity to predict upper‐limb outcome at 3 months (Stinear et al. 2017). In patients with substantial early upper‐limb impairment, the absence of an elicitable MEP identifies a subgroup with poorer expected outcomes and triggers further stratification according to stroke severity or structural lesion burden, whereas preserved MEPs indicate residual corticospinal integrity and are associated with a more favorable prognosis. Importantly, MEP absence should be interpreted as a strong marker of impaired corticospinal reserve rather than as a deterministic indicator of poor recovery, because outcome also depends on clinical severity, lesion characteristics, age, rehabilitation exposure, and other biological factors (Stinear et al. 2012; Byblow et al. 2015).

However, the prognostic value of MEP status should not be conflated with its potential role as a treatment‐response modifier. A prognostic marker is associated with subsequent outcome irrespective of the treatment received, whereas a treatment‐response modifier identifies whether the relative effect of an intervention differs according to baseline marker status (Stinear 2017; Boyd et al. 2017). Demonstrating the latter requires a formal treatment‐by‐marker interaction analysis rather than separate within‐subgroup significance testing. Because the treatment‐response interpretation in the present review relies predominantly on one randomized trial (Qian et al. 2024), MEP status should currently be regarded as a promising baseline stratification measure rather than a validated predictor of differential response to rTMS. This interpretation is also consistent with recent evidence showing that baseline MEP status did not predict response to upper‐limb training in individuals with chronic moderate‐to‐severe post‐stroke hemiparesis (King et al. 2025). Furthermore, MEP‐negative status may reflect severe corticospinal dysfunction, but it can also be influenced by the target muscle, stimulation intensity, lesion location, recording conditions, and timing of assessment (Rossini et al. 2015; Groppa et al. 2012).

Treatment‐related RMT changes accompanied motor improvement in some trials but were absent in others despite clinical benefit (Chen et al. 2021; Qian et al. 2024; Guan et al. 2017; Liao et al. 2024). RMT reflects the excitability of the neural elements required to produce a minimally detectable peripheral response and is strongly influenced by membrane excitability and axonal recruitment. It does not directly quantify synaptic efficacy, motor‐network connectivity, or the capacity to generate coordinated movement. RMT may consequently be useful for describing corticospinal excitability and determining stimulation dose, but its isolated use as a recovery marker is limited. It is also important to distinguish trials in which RMT was analyzed as an outcome from those in which it was used only to set treatment intensity.

The evidence for intracortical inhibitory measures was substantially more limited. Liang et al. (2024) reported reduced SICI after combined central and peripheral magnetic stimulation, suggesting modulation of GABA_A_‐related intracortical inhibition, but this change was not significantly related to functional gains. Lüdemann‐Podubecká et al. (2016) found clinical improvement without changes in CSP, whereas other measures such as ICF and LICI were rarely or not consistently reported. This variability is biologically plausible because intracortical inhibition changes across the recovery trajectory and may have different functional meanings according to impairment severity and motor state. McDonnell and Stinear (2017) found that ipsilesional SICI was generally reduced early after stroke but was not consistently different from control values in the chronic phase. In chronic, well‐recovered patients, reduced SICI has been associated with greater pinch strength, suggesting that disinhibition may support residual motor output in some individuals (Ferreiro de Andrade and Conforto 2018).

An important source of heterogeneity across TMS‐derived measures is the behavioral and physiological state in which they are acquired. This issue is not restricted to SICI or IHI. MEP amplitude is influenced by background muscle activation, motor preparation, voluntary contraction, attentional state, and the timing of stimulation relative to movement, such that responses obtained at rest may differ substantially from those elicited during active motor states (Rossini et al. 2015; Groppa et al. 2012; Bestmann and Krakauer 2015). Similarly, intracortical inhibitory and facilitatory measures are dynamically modulated during movement preparation and execution. In stroke populations, movement‐related SICI abnormalities may persist even when resting measurements are less informative, suggesting that task‐related assessments can reveal physiological deficits that are not evident under resting conditions (Ding et al. 2018). Interhemispheric interactions are also state dependent, with the magnitude and direction of inhibition varying according to whether measurements are obtained at rest, during tonic contraction, or in relation to an upcoming movement (Mirdamadi et al. 2023). Therefore, direct comparison across studies requires careful consideration of the motor state, background EMG activity, task demands, and timing of stimulation relative to behavior. Failure to account for these factors may contribute to apparently conflicting neurophysiological findings across stroke studies.

Interhemispheric measures were similarly heterogeneous. The classical interhemispheric competition model proposes that excessive inhibitory influence from the contralesional hemisphere may suppress ipsilesional motor output and contribute to impairment. However, the seminal findings of Murase et al. should be interpreted in the context of motor state. In that study, IHI from the contralesional to the ipsilesional M1 was not abnormally increased at rest; rather, the critical abnormality emerged during movement preparation, close to the onset of voluntary movement, when inhibition was normally released in healthy participants but remained prominent in individuals with chronic stroke (Murase et al. 2004). Thus, the rationale for suppressing contralesional excitability with low‐frequency rTMS was partly derived from a state‐specific abnormality rather than from a stable resting interhemispheric imbalance.

Importantly, the functional significance of contralesional activity may also depend on the residual structural and physiological capacity of the ipsilesional motor system. Although the classical interhemispheric competition model provided the rationale for facilitating ipsilesional M1 or suppressing contralesional M1 after stroke, this framework does not appear to apply uniformly across patients (Di Pino et al. 2014; Hummel and Cohen 2006). The bimodal balance‐recovery model proposes that the influence of the contralesional hemisphere depends on ipsilesional structural reserve, particularly the integrity of residual corticospinal and motor pathways. In individuals with relatively preserved structural reserve, excessive contralesional influence may be maladaptive and interfere with ipsilesional motor output, whereas in patients with more extensive corticospinal damage and lower structural reserve, recruitment of contralesional motor regions may serve a compensatory role (Di Pino et al. 2014; Lin et al. 2020). Subsequent experimental studies have challenged a strict interhemispheric competition model, showing that increasing contralesional excitability may not necessarily impair motor performance in more severely affected individuals and that the relationship between contralesional inhibition, impairment, and corticospinal reserve is nonlinear (Lin et al. 2020; Xu et al. 2019; McCambridge et al. 2018). These observations suggest that the direction and clinical relevance of interhemispheric interactions should be interpreted in relation to lesion severity, corticospinal integrity, and residual motor‐system reserve rather than according to a uniform model of pathological contralesional overactivity.

Subsequent evidence supports the view that IHI is dynamically modulated by motor state. Reviews of the field have emphasized that discrepancies among stroke studies may partly reflect differences in the movement phase during which IHI is measured and that abnormalities may be most apparent during movement initiation rather than at rest (Boddington and Reynolds 2017). More recent work in chronic stroke has likewise shown that state‐dependent modulation of IHI between rest and voluntary contraction is more closely related to motor impairment than resting IHI alone (Mirdamadi et al. 2023). However, the contemporary randomized‐trial evidence does not yet demonstrate that neuromodulation studies using movement‐related or pre‐movement IHI measurements consistently yield superior clinical or neurophysiological outcomes. Rather, these findings suggest that failure to account for behavioral state may obscure physiologically relevant abnormalities and contribute to heterogeneity across studies. Future trials should therefore consider incorporating both resting and task‐ or movement‐related IHI assessments when evaluating interhemispheric mechanisms and treatment response.

Measurement reliability is an additional concern. Paired‐coil IHI requires stable test MEPs, which may be difficult or impossible to obtain from the affected hemisphere in severely impaired patients. A pilot reliability study demonstrated that the reproducibility of IHI indices after stroke can vary substantially, limiting their use as longitudinal endpoints in small trials (Cassidy et al. 2016). TMS combined with EEG may help overcome some of these limitations by measuring cortical responses without requiring a recordable peripheral MEP. Casula et al. demonstrated that TMS–EEG could identify interhemispheric imbalance in patients without an evocable MEP from the affected hemisphere and linked these measures with callosal integrity and hand recovery. These approaches may extend physiological assessment to severely affected participants who are systematically underrepresented in conventional MEP‐based studies (Casula et al. 2021).

One of the main observations of this review was that clinical improvement frequently occurred without a corresponding change in the selected TMS measure. This should not be interpreted as evidence that the intervention lacked a physiological effect. Rather, conventional TMS may assess only a limited component of the neural changes supporting recovery. Clinical scales can improve through restitution of impaired movements, compensatory strategies, increased strength, altered movement speed, improved balance, learning, or greater independence. These processes may depend on different neural systems and may not produce parallel changes in a single MEP, threshold, or inhibitory variable. Conversely, a statistically significant neurophysiological change should not automatically be assumed to represent meaningful functional recovery.

A further interpretative issue is that most included interventions were not delivered in isolation. Neuromodulation was commonly combined with physical therapy, upper‐limb training, conventional rehabilitation, or motor‐task practice. Therefore, within‐group improvement may reflect the combined effects of spontaneous recovery, repeated motor training, rehabilitation exposure, and stimulation. This is particularly relevant in acute and subacute stroke, when substantial biological recovery may occur independently of the experimental intervention. Only between‐group comparisons against sham stimulation or rehabilitation‐matched control conditions can estimate the incremental contribution of rTMS or TBS. Across the included trials, some studies demonstrated superior motor outcomes with active stimulation, whereas others showed similar clinical improvement despite absent or nonspecific neurophysiological differences. Thus, the present evidence supports interpreting rTMS primarily as a potential adjunct to rehabilitation rather than as an independent determinant of motor recovery.

These considerations also emphasize the importance of distinguishing association from mediation. A correlation between change in MEP amplitude and change in FMA‐UE does not establish that the electrophysiological change caused the clinical improvement. Both may reflect a third process, such as spontaneous biological recovery, concurrent motor training or rehabilitation exposure, lesion characteristics, or baseline corticospinal integrity. To establish a mechanistic biomarker, future trials should prespecify causal hypotheses, measure the proposed mediator at appropriate time points, report between‐group effects, and, when feasible, perform longitudinal mediation analyses. Candidate treatment‐response modifiers should be examined using interaction terms rather than separate significance tests within biomarker‐defined subgroups.

From a clinical perspective, MEP status and MEP amplitude currently appear to be the most informative TMS‐derived measures identified by this review, but their roles differ. Baseline MEP status may contribute to prognosis and participant stratification, whereas longitudinal MEP amplitude may provide a measure of treatment‐related corticospinal change. RMT offers complementary information but appears less sensitive and should not be interpreted as an independent marker of functional recovery. SICI, CSP, iSP, and IHI remain valuable for mechanistic investigation, but their clinical application is restricted by limited evidence, task dependence, technical complexity, and uncertain reliability.

The methodological comparison across the included trials further illustrates why a single TMS‐derived measure cannot yet be interpreted uniformly across studies. Significant and null neurophysiological findings occurred under overlapping stimulation and acquisition conditions, and no single methodological feature consistently characterized studies showing a neurophysiological–behavioral association. Longitudinal changes in MEP amplitude were the most recurrent physiological finding accompanying clinical improvement, particularly in upper‐limb studies, but this pattern was not universal. Differences in target muscle, hemisphere assessed, test intensity, number of trials, motor state during recording, assessment timing, stroke stage, baseline corticospinal integrity, and concurrent rehabilitation are therefore more likely to act jointly than as isolated determinants of whether a significant TMS effect is detected. Incomplete reporting of several of these parameters further limits identification of reproducible methodological predictors of positive findings. On the basis of this cross‐study methodological heterogeneity, future randomized trials should use standardized TMS procedures and report coil type and orientation, stimulation waveform, target localization, neuronavigation, target muscle, background electromyographic activity, threshold definition, stimulation intensity, number of accepted trials, MEP exclusion criteria, averaging method, hemisphere assessed, recording state, and assessment timing. Studies should also report neurophysiological results separately for MEP‐positive and MEP‐negative participants and account for stroke stage, lesion location, impairment severity, medication use, and concurrent rehabilitation. Multimodal protocols integrating TMS with diffusion imaging, functional neuroimaging, EEG, or biomechanical measures may provide a more complete representation of recovery than isolated corticospinal variables. Such standardization is consistent with recommendations that stroke biomarkers be biologically plausible, reliable, reproducible, clinically feasible, and evaluated for a clearly prespecified purpose (Boyd et al. 2017).

Several limitations should be considered. First, restricting inclusion to randomized controlled trials reduced the number of eligible studies, particularly for SICI, ICF, CSP, iSP, and IHI. The review was also restricted to train‐based rTMS and TBS paradigms; therefore, associative TMS approaches lacking an eligible train‐based rTMS or TBS component were not included. These paradigms may provide complementary information regarding post‐stroke network plasticity but involve distinct stimulation principles and should be evaluated separately in future syntheses. Second, the restriction to studies published from 2016 onward limits the review to contemporary randomized evidence and may have excluded relevant earlier trials of post‐stroke rTMS. Accordingly, the present findings should not be interpreted as a synthesis of the entire historical literature. Third, substantial heterogeneity was observed across studies in stroke stage, baseline impairment, lesion characteristics, stimulation target, treatment dose, concurrent rehabilitation, comparator condition, and follow‐up duration. Fourth, TMS acquisition and analysis procedures varied considerably, limiting direct cross‐study comparisons and the identification of reproducible neurophysiological patterns. Fifth, several neurophysiological outcomes were secondary or exploratory and may therefore have been more susceptible to selective reporting. In addition, improvements observed within active‐treatment groups were not always accompanied by significant between‐group differences, limiting causal interpretation of treatment‐related neurophysiological change. Finally, heterogeneity in outcome definitions, acquisition methods, and statistical approaches precluded meta‐analysis, and the resulting narrative synthesis cannot provide pooled estimates of association or treatment‐related effects.

Overall, MEP‐derived measures provide the most developed neurophysiological evidence in the current randomized‐trial literature, but they should be interpreted within a multimodal and purpose‐specific framework. Baseline MEP status is better supported as a measure of corticospinal integrity and recovery potential than as a validated predictor of differential rTMS response. MEP amplitude may help monitor corticospinal change, whereas RMT and inhibitory measures provide complementary but less consistent information. Larger multicenter trials with harmonized acquisition protocols, prespecified biomarker hypotheses, repeated measurements, and formal treatment‐by‐biomarker analyses are needed before TMS‐derived measures can reliably guide individualized post‐stroke neuromodulation.

## 5 Conclusion

This systematic review found that MEP amplitude, MEP latency, and RMT are the most frequently reported TMS‐derived measures associated with post‐stroke motor recovery. Direct randomized‐trial evidence for ICF and LICI was absent, whereas evidence for SICI, CSP, iSP, and IHI was very limited and inconsistent. These findings support the use of TMS‐derived measures primarily as complementary tools for monitoring corticospinal changes rather than as established predictors of recovery. Future trials should standardize acquisition methods, prespecify neurophysiological outcomes, and evaluate whether baseline TMS profiles can reliably stratify treatment response.

## Author Contributions

**Gabrielly Santos Pereira:** conceptualization, investigation, writing – original draft, methodology, validation, visualization, writing – review and editing, software, formal analysis, data curation. **Marcelo Lourenço da Silva:** conceptualization, investigation, methodology, validation, visualization, writing – review and editing, software, formal analysis, data curation. **Abrahão Fontes Baptista:** conceptualization, investigation, writing – original draft, methodology, validation, visualization, writing – review and editing, data curation. **João Eduardo de Araujo:** conceptualization, investigation, funding acquisition, writing – original draft, methodology, validation, visualization, writing – review and editing, software, formal analysis, data curation, supervision, resources, project administration.

## Funding

This study was supported by the Coordenação de Aperfeiçoamento de Pessoal de Nível Superior—Brazil (CAPES; Grant 88887.254059/2026‐00).

## Conflicts of Interest

The authors declare no conflicts of interest.

## Supporting information


**Table S1:** Methodological characteristics of TMS‐derived neurophysiological assessments in the included randomized controlled trials.

## Data Availability

All data supporting this systematic review were obtained from published studies cited in the manuscript. Additional extraction and appraisal files are available from the corresponding author upon reasonable request.
