# Supplementary material for: TMS‐Derived Neurophysiological Measures of Corticospinal Excitability, Intracortical Excitability, and Interhemispheric Interactions Associated With Post‐Stroke Motor Recovery: A Systematic Review
Source: Eur J Neurosci. 2026 Oct 4;64(7):e70698. doi: 10.1111/ejn.70698 (PMC13634656; doi:10.1111/ejn.70698)
Supplement: Supplementary file 1 [file EJN-64-0-s001.docx]

**Supplementary Table S1. Methodological characteristics of TMS-derived neurophysiological assessments in the included randomized controlled trials**

| **Study** | **Target muscle / hemisphere assessed** | **Coil / localization** | **Motor-threshold definition** | **MEP acquisition / number of trials** | **Recording state and EMG/processing** | **Assessment timing** |
| --- | --- | --- | --- | --- | --- | --- |
| **Ackerley et al.** | Bilateral FDI; ipsilesional and contralesional M1 | Individual FDI hotspot; single-pulse TMS | RMT: ≥50 μV peak-to-peak in 4/8 trials. AMT: >100 μV in 4/8 trials during ~20% MVC | Stimulus-response curves using 12 MEPs per intensity at −5%, +5%, +15%, +25%, and +35% MSO relative to RMT; 4–5-s interstimulus interval | Surface EMG; resting FDI for RMT/MEPs; trials rejected if pre-stimulus rmsEMG >10 μV; peak-to-peak MEPs averaged | Two baselines; mid-intervention; immediate post; 1 month; 3 months |
| **Du et al.** | APB; bilateral hemispheres | Figure-of-eight coil; M1 hotspots | Exact diagnostic RMT definition NR | Bilateral RMT and MEP amplitude/latency; test intensity and number of accepted MEPs NR | EMG methodology incompletely reported in accessible text | Baseline; after 5 sessions; 3-month follow-up |
| **Lüdemann-Podubecká et al.** | Bilateral FDI | M1 hand area; contralesional PMd stimulation site referenced to FDI hotspot | RMT: ≥50 μV in ≥5/10 trials | 20 MEPs at 110% RMT | Hands relaxed; peak-to-peak MEP amplitudes calculated offline and averaged. CSP obtained during active FDI contraction | Immediately before and after each real/sham crossover session |
|  |  |  |  |  |  |  |
| **Guan et al.** | Bilateral ADM | Figure-of-eight MC-B70-type configuration; M1 | Lowest intensity producing ≥5 MEPs of 50 μV peak-to-peak in 10 trials | RMT was the principal neurophysiological endpoint; separate MEP-amplitude protocol not reported | Resting muscle for threshold determination | Pretreatment; second day post-treatment; 1, 3, 6, and 12 months |
| **Huang et al.** | Quadriceps/rectus femoris representation; leg motor cortex | 110-mm double-cone coil | AMT used; exact response criterion NR | MEP amplitude and latency assessed; test intensity and number of trials NR | Recording/processing details NR | Baseline; post-intervention; 3-month follow-up |
| **El-Tamawy et al.** | FDI; both hemispheres | Magstim Rapid²; 70-mm figure-of-eight coil | cAMT/iAMT determined from the minimum intensity producing observable contralateral FDI activation; detailed EMG threshold criterion NR | MEP waveform amplitude was not the principal outcome; no standardized MEP trial series reported | AMT-based cortical excitability assessment; detailed waveform processing NR | Pre- and post-intervention |
| **Meng et al.** | Target muscle NR; ipsilesional corticospinal output emphasized | M1; coil/acquisition configuration NR | NR | MEP amplitude and latency assessed before and after treatment; stimulation intensity and number of trials NR | EMG processing and background activity criteria NR | Baseline and after 10 treatment sessions |
| **Chen et al.** | Target muscle NR; affected-hemisphere excitability emphasized | Bilateral M1; acquisition configuration NR | RMT assessed; detailed threshold criterion NR | MEP amplitude and CMCT reported; test intensity and number of trials NR | Detailed EMG/MEP processing NR | 24 h before and 24 h after the 4-week intervention; clinical follow-up at 3 months |
| **Luk et al.** | Bilateral FDI | M1 hotspot with neuronavigation | RMT: ≥50 μV in ≥5/10 responses; MEP absent if no response at 100% MSO | 10 MEPs averaged at 120% baseline RMT; 100% MSO used when paretic MEP remained absent | Surface EMG; peak-to-peak amplitude; background activation controlled; acquisition sampled at high frequency | Baseline; 2 weeks; 12 weeks |
| **Lv et al.** | APB; contralesional stimulation hotspot; serial excitability assessment | M1 hotspot | RMT based on responses in >5/10 trials; the article reports an unusually high amplitude unit (“50 mV”), so the criterion should be interpreted cautiously | MEP amplitude reassessed after successive 10-session blocks; acquisition intensity and number of MEPs NR | Resting threshold assessment; detailed processing NR | Baseline and after the treatment blocks corresponding to 10, 20, or 30 sessions |
| **Liang et al.** | APB; lesional M1; mirror hotspot used when no affected-side MEP could be elicited | Central M1 TMS; peripheral stimulation over paretic C7 nerve root | RMT: ≥50 μV in ≥5/10 consecutive stimuli | MEP: 120% RMT; 10 successive stable waveforms averaged. SICI: conditioning pulse 70% RMT, test pulse at MEP intensity, 2-ms ISI | Measurements obtained at rest; peak-to-peak MEP amplitude and latency; SICI expressed relative to single-pulse MEP | Baseline and post-intervention |
| **Liao et al.** | FDI; ipsilesional M1 for corticospinal assessment | M1 and contralesional cerebellar stimulation conditions | RMT: minimum intensity eliciting ≥50 μV in ≥5/10 responses in relaxed FDI | MEP amplitude and CSP assessed; MEP test intensity and number of accepted trials NR | RMT obtained at rest; detailed MEP averaging/CSP processing NR | Baseline; during intervention; end of 3-week treatment; follow-up |
| **Qian et al.** | Bilateral APB | Bilateral M1; single-channel EMG/MEP monitoring | RMT: ≥50 μV in ≥5/10 responses. MEP-positive status: reproducible MEP ≥200 μV in ≥2/3 responses under the specified protocol | 10 MEPs averaged at 120% baseline RMT; CMCT additionally assessed using cervical stimulation | APB primarily relaxed; slight voluntary activation could be used during MEP-status determination when required; digital EMG acquisition | Baseline; 4 weeks; 8 weeks |
| **Valovičová et al.** | FDI; ipsilesional M1; mirror of contralesional hotspot when no ipsilesional MEP | Neuro-MS/D system; cooled angulated figure-of-eight coil | RMT assessed; detailed response criterion NR | MEP amplitude, latency, and CMCT; test intensity and number of accepted trials NR | Surface EMG; detailed processing criteria NR | Baseline during acute hospitalization; after completion of 5 rTMS sessions |
| **Zhao et al.** | Target muscle NR | Figure-of-eight coil; M1 localized from CPz–C3/C4 midpoint; SMA at FC1/FC2 | RMT used for dosing; diagnostic threshold definition NR | TMS-MEP amplitude and latency assessed; test intensity and number of trials NR | Detailed EMG processing and background muscle state NR | Before and after 4-week intervention |
| **Li et al.** | Bilateral EDC for cortical/IHI assessment; paretic biceps brachii for ipsilateral MEP assessment | Magstim single-pulse system with figure-of-eight coil; MRI-guided Brainsight neuronavigation | AMT obtained during 20 ± 5% MVC; response required MEP >100 μV above background in ≥6/10 trials | IHI/iSP: stimulation at 100% MSO during ~50% MVC, 40 trials to obtain ≥15 clear iSPs. Ipsilateral MEP: 100% MSO, 40 trials to obtain ≥10 clear responses | Surface EMG; bilateral EDC sampled at 4 kHz and band-pass filtered; iSP onset/offset determined using predefined signal criteria | Baseline and end of 6-week intervention; motor outcomes also followed at 3 months |

**Supplementary Table S1 note:** NR, not reported or not sufficiently specified in the original report; AMT, active motor threshold; APB, abductor pollicis brevis; CMCT, central motor conduction time; CSP, cortical silent period; EDC, extensor digitorum communis; EMG, electromyography; FDI, first dorsal interosseous; IHI, interhemispheric inhibition; ISI, interstimulus interval; MEP, motor evoked potential; MSO, maximum stimulator output; MVC, maximum voluntary contraction; RMT, resting motor threshold; SICI, short-interval intracortical inhibition.
